# Histological validation of cardiovascular magnetic resonance T1 mapping markers of myocardial fibrosis in paediatric heart transplant recipients

**DOI:** 10.1186/s12968-017-0326-x

**Published:** 2017-02-01

**Authors:** Seiko Ide, Eugenie Riesenkampff, David A. Chiasson, Anne I. Dipchand, Paul F. Kantor, Rajiv R. Chaturvedi, Shi-Joon Yoo, Lars Grosse-Wortmann

**Affiliations:** 1 Division of Cardiology, Department of Paediatrics, Labatt Family Heart Centre, The Hospital for Sick Children, University of Toronto, 555 University Avenue, Toronto, ON M5G 1X8 Canada; 2 Division of Pathology, Department of Paediatric Laboratory Medicine, The Hospital for Sick Children, University of Toronto, Toronto, ON Canada; 3 Division of Cardiology, Department of Paediatrics, Stollery Children’s Hospital, Edmonton, AB Canada; 4 Department of Diagnostic Imaging, The Hospital for Sick Children, University of Toronto, Toronto, ON Canada

**Keywords:** Paediatric heart transplantation, Diffuse myocardial fibrosis, Cardiovascular magnetic resonance, Native T1 time, Extracellular volume fraction, Histological validation

## Abstract

**Background:**

Adverse fibrotic remodeling is detrimental to myocardial health and a reliable method for monitoring the development of fibrotic remodeling may be desirable during the follow-up of patients after heart transplantation (HTx). Quantification of diffuse myocardial fibrosis with cardiovascular magnetic resonance (CMR) has been increasingly applied and validated histologically in adult patients with heart disease. However, comparisons of CMR findings with histological fibrosis burden in children are lacking. This study aimed to compare native T1 times and extracellular volume fraction (ECV) derived from CMR with the degree of collagen on endomyocardial biopsy (EmBx), and to investigate the association between myocardial fibrosis and clinical as well as functional markers in children after HTx.

**Methods:**

EmBx and CMR were performed on the same day. All specimens were stained with picrosirius red. The collagen volume fraction (CVF) was calculated as ratio of stained collagen area to total myocardial area on EmBx. Native T1 values and ECV were measured by CMR on a mid-ventricular short axis slice, using a modified look-locker inversion recovery approach.

**Results:**

Twenty patients (9.9 ± 6.2 years of age; 9 girls) after HTx were prospectively enrolled, at a median of 1.3 years (0.02–12.6 years) post HTx, and compared to 24 controls (13.9 ± 2.6 years of age; 12 girls). The mean histological CVF was 10.0 ± 3.4%. Septal native T1 times and ECV were higher in HTx patients compared to controls (1008 ± 32 ms vs 979 ± 24 ms, *p* < 0.005 and 0.30 ± 0.03 vs 0.22 ± 0.03, *p* < 0.0001, respectively). CVF showed a moderate correlation with native T1 (*r* = 0.53, *p* < 0.05) as well as ECV (*r* = 0.46, *p* < 0.05). Native T1 time, but not ECV and CVF, correlated with ischemia time (*r* = 0.46, *p* < 0.05).

**Conclusions:**

CMR-derived fibrosis markers correlate with histological degree of fibrosis on EmBx in children after HTx. Further, native T1 times are associated with longer ischemia times.

**Electronic supplementary material:**

The online version of this article (doi:10.1186/s12968-017-0326-x) contains supplementary material, which is available to authorized users.

## Background

In spite of substantially improved survival following paediatric heart transplantation (HTx) over the past two decades [1], significant morbidity still prevails, some of it limiting the survival of the graft organ [1, 2].

Ventricular dysfunction in HTx recipients has been linked to previous or acute episodes of organ rejection and to coronary allograft vasculopathy (CAV). Both rejection and CAV are suspected to cause myocardial fibrotic remodeling [3–5] which, in turn, results in ventricular dysfunction [6, 7]. Ultimately, adverse fibrotic remodeling is detrimental to myocardial health [8, 9]. Since the development of fibrosis has implications for long term graft function and survival [10, 11], a non-invasive reliable method to monitor graft fibrosis may be clinically useful during the follow-up of patients at risk, including HTx recipients.

Histological evaluation of tissue is the gold standard and has been the only available method for quantification of myocardial fibrosis. However, its invasive nature and the exposure to ionizing radiation during cardiac catheterization represent significant barriers to serial endomyocardial biopsies (EmBxs), especially in children. Furthermore, the biopsied region may not be representative of the majority of the myocardium and may be affected by scarring due to previous sampling in the same location. More recently, cardiovascular magnetic resonance (CMR) techniques based on T1 relaxometry have been refined and applied in adults with a variety of cardiac diseases, including in HTx recipients [12–20]. CMR-derived markers include native T1 times and extracellular volume fraction (ECV), both of which have been validated histologically in adults [16, 21–26], and both of which are predictive of heart failure onset and mortality in adults with ischemic and non-ischemic cardiomyopathy [27, 28]. However, neither native T1 nor ECV has been histologically validated against EmBx in children and adolescents. We hypothesized that native T1 and ECV are associated with the histological degree of fibrosis within the myocardium of pediatric HTx recipients.

The aims of this study in children after HTx were 1) to compare CMR native T1 times and ECV with the degree of fibrosis on histological samples and 2) to investigate the association between the histological degree of fibrosis and CMR-derived fibrosis markers on the one hand and markers of cardiac health and transplant-related factors on the other.

## Methods

### Study protocol and population

Following approval by the Hospital for Sick Children Research Ethics Board (project # 1000013662), all HTx children and adolescents who were scheduled for a clinical EmBx for rejection surveillance in asymptomatic patients between April 2010 and October 2011 were invited to participate in the study. Following informed written consent by the patients and/or their legal guardian(s), patients underwent a research CMR immediately prior to the catheterization with the EmBx procedure.

CMR studies were also performed in 24 controls, consisting of children with a family history of arrhythmogenic right ventricular cardiomyopathy or with non-specific chest pain who were referred for anatomical coronary artery imaging. Only children who had an entirely normal CMR examination as well as normal results on all other tests were included.

### Magnetic resonance image acquisition

The CMR examinations were performed using a 1.5 Tesla system (‘Avanto’, Siemens Healthcare Sector, Erlangen, Germany). Ventricular volumes were assessed with a stack of multiphase short axis slices, acquired with a steady-state free-procession (SSFP) sequence in the routine clinical fashion [29]. A modified Look-Locker inversion recovery (MOLLI) sequence was used to measure native and postcontrast longitudinal relaxation T1 times of myocardium and within the blood pool of the LV cavity as has been described [30]. The sequence included two inversion-recovery prepared, electrocardiogram synchronized Look-Locker experiments with inversion pulses of 100 ms and 150 ms, respectively, with three and five single-shot images after each of these inversion pulses. Other imaging parameters were as follows: Repetition and echo times 2.53 ms and 1.08 ms, respectively; in-plane resolution, 1.7 × 1.7 mm; slice thickness, 8 mm; flip angle, 35°.

Images were acquired in diastole at a single mid-ventricular short axis slice before and 10 min after the administration of 0.2 mmol/kg gadopentetate dimeglumine (‘Magnevist’, Bayer, Leverkusen, Germany). Breathholds were used in awake and cooperative patients; all other patients and anaesthetized patients were scanned during free breathing or ventilation. The presence of late gadolinium enhancement (LGE) was assessed qualitatively on long and short-axis phase sensitive inversion recovery images in the usual clinical fashion.

### Magnetic resonance image analysis

#### T1 times and ECV

**Fig. 1:**
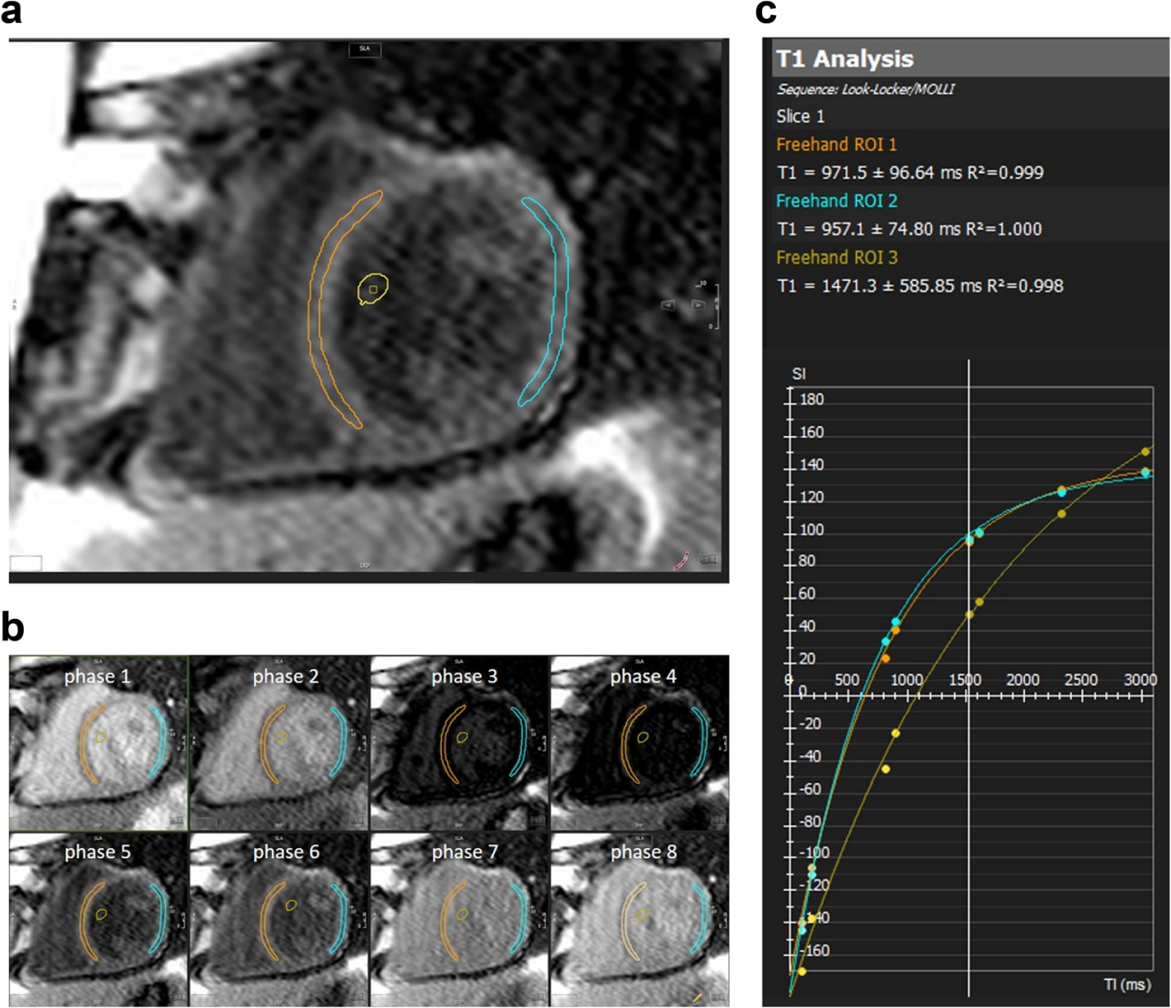
Quantification of T1 times. Regions of interest were drawn within the blood pool (*yellow*), the interventricular septum (*orange*), left ventricular free wall (*blue*), entire left ventricular circumference (not shown) (panel **a**) and blood pool on each of the 8 source images (panel **b**), taken at different inversion times. A curve fitting algorithm computed the T1 times for each region of interest (panel **c**)

Longitudinal relaxation times (T1 times) were measured using a curve-fitting algorithm within commercially available software (CVI42, Circle Cardiovascular Imaging, Calgary, Canada). Regions of interest were drawn manually on the raw images of MOLLI mid-ventricular short axis slices to represent the interventricular septum (IVS) (equivalent to segments 8 and 9 of the American Heart Association 17 segments left ventricular (LV) model [31]) and the entire LV myocardial circumference (representing segments 7–12). To avoid partial volume effects at myocardial borders, regions of interest were confined to the central two thirds of the myocardium, and were manually adjusted on each raw image in order to compensate for inter-image variability in diaphragm position (Fig. 1). Pre- and postcontrast blood T1 times were measured in regions of interest manually drawn in the center of the LV cavity so as to avoid trabeculations or papillary muscles (Fig. 1). A second observer (LGW), who was blinded towards the results by observer 1 (ER), measured pre- and postcontrast T1 within the IVS and blood. Extracellular volume fraction was calculated from pre- and postcontrast T1 times for each region as well as the hematocrit [15] which was obtained on the same day according to the established formula [16].

#### Feature tracking

Feature tracking analysis was performed with the TomTec 2D CPA MR software (TomTec Imaging Systems, Unterschleissheim, Germany) using standard SSFP cine images, with 20 phases per cardiac cycle, as previously described [32]. In brief, endocardial borders were manually drawn in the end-diastolic frame, and then were automatically propagated through the cardiac cycle. The four-chamber view cines were tracked to derive global LV longitudinal strain and strain rate, while short-axis cines were used to derive global LV circumferential and radial strain and strain rate in the same location as the T1 and ECV measurements, i.e. at the level of the papillary muscles of the LV.

#### Ventricular volumes and function

Ventricular volumes were obtained from the cine short axis stack using dedicated software (QMass, version 7.2; Medis, Leiden, The Netherlands) according to the Society for Cardiovascular MR guidelines for reporting cardiovascular MR examinations [33]. Ventricular volumes and LV mass were indexed by body surface area and ejection fractions were calculated from enddiastolic and endsystolic ventricular volumes.

### Histological analysis

**Fig. 2:**
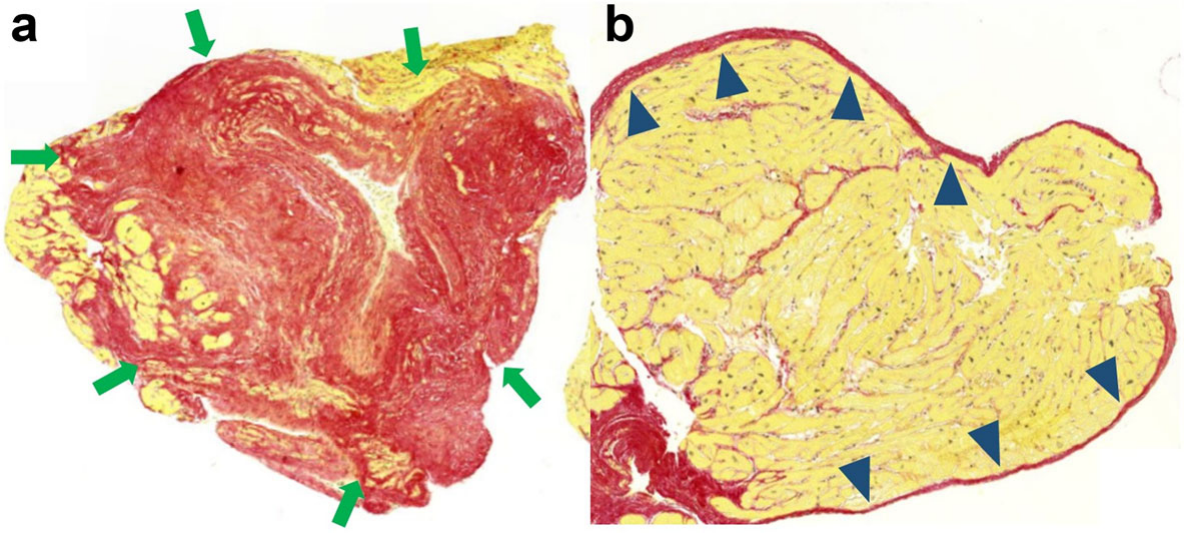
Examples of areas excluded from histological analysis. **a** The red stained area delineated with green arrows was estimated as scaring from a previous biopsy and excluded from analysis. **b** Endocardial fibrous tissue, indicated with blue arrow heads, was also excluded from analysis

Transvenous right ventricular biopsies were obtained from the right ventricular aspect of the IVS immediately following the CMR in the routine clinical fashion. Three to six samples were taken in each individual. Samples (100 in all) were immediately fixed in 10% buffered formalin, processed and embedded in paraffin, and serially sectioned, with every second slide stained with hematoxylin and eosin as per standard protocol. A mid-level unstained section was selected and stained with picrosirius red for the analysis of collagen. Stained sections were digitalized with a Pannoramic 250 Flash IIslide scanner (3DHISTECH, Budapest, Hungary). Low-power views were was used to select appropriate areas for analysis, excluding superficial endocardial fibrous tissue and sites of scarring from previous biopsies at the discretion of the interpreting pathologist (Fig. 2). Specimens were photographed at ×20 magnification in all appropriate myocardial regions.

**Fig. 3:**
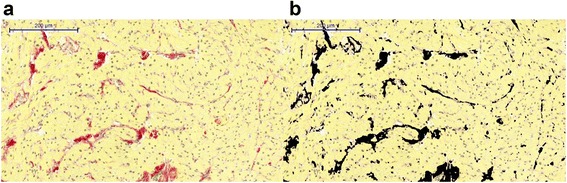
Calculation of collagen volume fraction. **a** Collagen is stained by picrosirius red on a myocardial specimen; the yellow counter stain represents myocytes. In a first step, all pixels in the image were counted. **b** In a second step, all white background pixels were counted and subtracted from the total myocardial area quantified in the image (**a**). After converting varying shades of red to black, using a thresholding algorithm, all black pixels were counted. This number was divided by the total number of pixels (minus the white ones) in the image to calculate the collagen volume fraction

The photographs were analyzed with automated image analysis software (macro written in ‘Image J’, National Institutes of Health, Bethesda, Maryland, USA), quantifying the total area of stained collagen and that of myocardium, excluding areas of white backgrounds in each sample (Fig. 3). This method for the assessment of collagen volume fraction (CVF) as a percent of the total tissue sampled has been shown to correlate well with the degree of fibrosis quantified by stereology and polarized light microscopy [34]. Collagen volume fraction was averaged among all biopsy specimens from each individual.

### Statistical analysis

Statistical analyses were performed using JMP pro version 11 software (SAS Institute Inc., Cary, North Carolina, USA). Data are presented as mean ± standard deviation or median with range, as appropriate; categorical variables are reported as number and percentage. Paired comparisons were performed by using Student t test or the Mann-Whitney U test for non-normally distributed data between patients and controls, and between groups of patients, where applicable. Linear regression analysis was performed by using the Pearson correlation coefficient for normally distributed data. Correlations were analyzed with use of Spearman’s rank correlation coefficient test for non-normally distributed data. Interobserver variability for native T1 times and ECV were assessed using the Bland-Altman method. All *p* values are two sided. Statistical significance was defined as p ≦ 0.05.

## Results

### Patient population

Patient characteristics are summarized in Table 1.

**Table 1:** Characteristics of patients and controls

	Patients	Controls	*p*
	(*n* = 20)	(*n* = 24)	
Male/female	11/9	12/12	0.74
Age at CMR, y	9.9 ± 6.2	13.9 ± 2.6	<0.05
Heart age at CMR, y	13.8 ± 9.3	13.9 ± 2.6	0.95
Body surface area, m^2^	1.19 ± 0.59	1.63 ± 0.28	<0.01
Heart rate at CMR, bpm	98 ± 12	76 ± 10	<0.0001
Hematocrit, %	36.9 ± 5.3	42.1 ± 3.6	<0.0005
NT-proBNP, pg/ml	49 (5–531)	NA	NA
Time since HTx at CMR, y	1.3 (0.02–12.6)	NA	NA
Ischemia Time, min^a^	207 ± 82 (*n* = 19)	NA	NA
Peak oxygen uptake (% of predicted)	66 ± 18 (*n* = 13)	NA	NA
Peak workload (% of predicted)	53 ± 5 (*n* = 13)	NA	NA

Data are expressed as mean and standard deviation except for time since HTx and NT-proBNP (reported in median and range)

*Abbreviations*: *NT-proBNP* N-terminal probrain natriuretic peptide, *CMR* cardiovascular magnetic resonance, *HTx* heart transplantation

^a^Data were available in 19 of 20 patients

Twenty patients (45% female) who had undergone HTx at a median age of 8.6 years (range, 0.02–16 years), and 24 controls were enrolled. Some of the results in a subgroup of patients were reported previously as part of a different study, exploring the association between CMR-derived fibrosis markers and cardiac function by echocardiography [17]. Patients were younger at the time of CMR than controls (*p* < 0.05). However, the organ age which was calculated as the sum of the donor age at demise and the time since HTx, was similar between the two groups. (Table 1) In terms of immunosuppressive medication, at the time of the CMR, 16 patients received tacrolimus and mycophenolate mofetil, two tacrolimus and mycophenolate mofetil in addition to prednisone, one tacrolimus monotherapy, and one sirolimus with mycophenolate mofetil. Three patients had previously taken Cyclosporine. Other medications included statins (*n* = 10) and amlodipine for arterial hypertension (*n* = 6). No patients were treated for diabetes mellitus. No patient had coronary angiography performed within 6 months of CMR.

### Imaging markers of fibrosis and histological validation

**Fig. 4:**
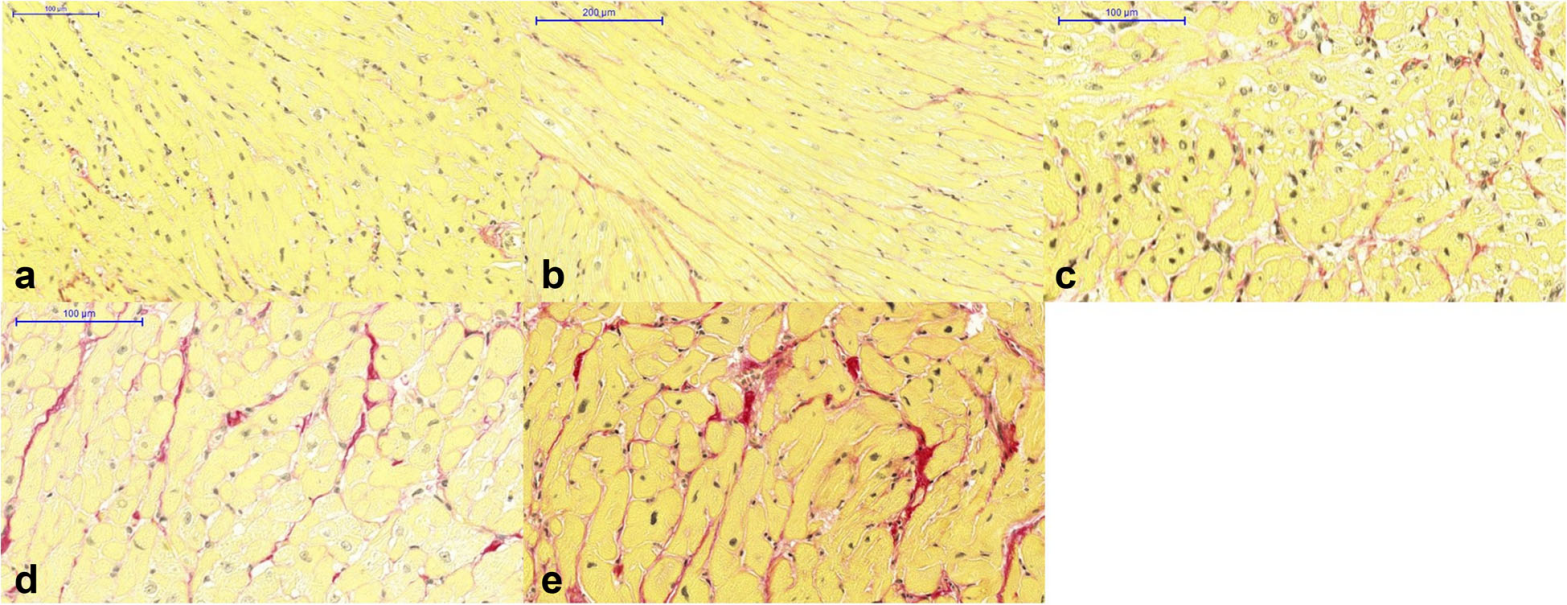
Examples of stained myocardial specimens from five patients. Tissue samples from intraventricular septum were stained with picrosirius red. Examples shown demonstrate **a**) 4.1%, **b**) 7.1%, **c**) 10.6%, **d**) 12.9% and **e**) 15.2% of collagen volume fraction (×20 magnification)

**Fig. 5:**
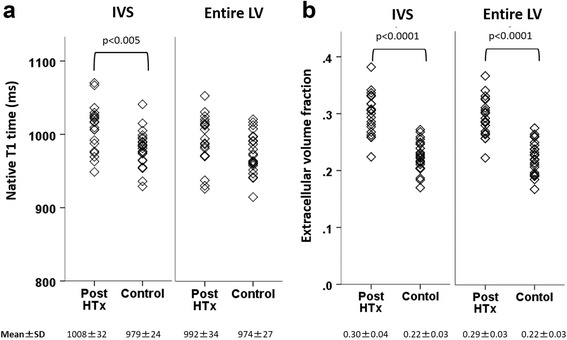
Difference of native T1 times and ECV between patients and controls. **a** Native T1 times were significantly higher in heart transplant (HTx) patients compared to controls in interventricular septum (IVS). The patient group showed significantly higher native T1 times in the IVS than in the left ventricular (LV) free wall. **b** The patients had significantly higher extracellular volume fraction (ECV) values in all investigated regions of LV myocardium as compared to controls

**Fig. 6:**
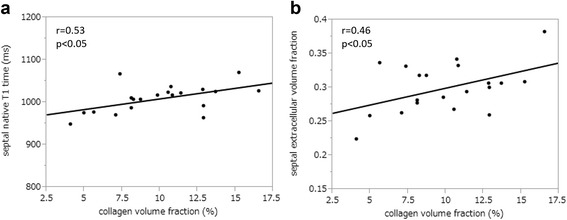
Correlation between collagen volume fraction and septal native T1 time or ECV Histological degree of fibrosis showed moderate correlation with septal native T1 times (**a**) as well as with septal extracellular volume fraction (ECV) (**b**)

Myocardial biopsy was collected without complication in all subjects. The mean histological CVF in all patients was 10 ± 3.4% (Fig. 4). The average CVF in all 100 biopsied specimens are shown in Additional file 1. MOLLI was acquired during breathholding in six patients (30%). Native T1 times and ECV were higher in HTx patients compared to controls within the IVS (*p* < 0.005 and *p* < 0.0001, respectively, Table 2) (Fig. 5a). Patients also had higher ECV values in the entire LV myocardium as compared to controls (*p* < 0.0001, Table 2, Fig. 5b). Histological CVF showed a moderate correlation with native T1 times as well as ECV within the IVS (*r* = 0.53, *p* < 0.05 and *r* = 0.46, *p* < 0.05, respectively, Fig. 6). There was no significant association between CVF and native T1 times or ECV within entire LV. Neither patients nor controls presented with evidence of focal scarring by LGE.

**Table 2:** Cardiovascular magnetic resonance parameters in patients and volunteers

		Patients	Controls	*p*
		(*n* = 20)	(*n* = 24)	
Native T1 time, ms	IVS	1008 ± 32	979 ± 24	<0.005
	Entire LV	992 ± 34	974 ± 27	0.06
	Blood	1515 ± 78	1537 ± 55	0.30
ECV	IVS	0.30 ± 0.04	0.22 ± 0.03	<0.0001
	Entire LV	0.29 ± 0.03	0.22 ± 0.03	<0.0001
LVEDVi, ml/m^2^		79 ± 17	88 ± 11	<0.01
LVESVi, ml/m^2^		37 ± 11	36 ± 7	0.62
LVEF, %		54 ± 5	59 ± 5	<0.005
LVMM, g/m^2^		65 ± 16	48 ± 12	<0.0005
LVMM/LVEDVi, g/ml		0.8 ± 0.2	0.5 ± 0.1	<0.0001
RVEDVi, ml/m^2^		89 ± 19	93 ± 12	0.38
RVESVi, ml/m^2^		47 ± 13	45 ± 8	0.45
RVEF, %		47 ± 8	52 ± 4	0.06
LV longitudinal strain, %		−13.4 ± 4.3^a^	−16.3 ± 4.8^b^	0.12
LV longitudinal strain rate, %/s		−1.0 ± 0.3^a^	−1.1 ± 0.4^b^	0.25
LV circumferential strain, %		−22.1 ± 4.0	−22.3 ± 2.9	0.88
LV circumferential strain rate, %/s		−1.5 ± 0.4	−1.4 ± 0.3	0.21
LV radial strain, %		25.7 ± 11.6	40.5 ± 9.9	<0.0001
LV radial strain rate, %/s		1.3 ± 0.3	1.8 ± 0.4	<0.0005

Data are expressed as mean and standard deviation

*Abbreviations*: *ECV* extracellular volume fraction, *IVS* interventricular septum; *LV* left ventricle, *LVEDV* left ventricular end-diastolic volume, *LVESV* left ventricular end-systolic volume, *LVEF* left ventricular ejection fractio, *LVMM* left ventricular muscle mass, *RVEDV* right ventricular end-diastolic volume, *RVESV* right ventricular end-systolic volume, *RVEF* right ventricular ejection fraction

^a^Data of LV longitudinal strain and strain rate were available in 19 of 20 patients

^b^Data of LV longitudinal strain and strain rate were available in 9 of 24 controls

### Interobserver variability

**Fig. 7:**
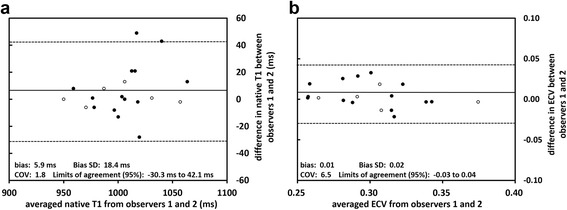
Bland-Altman plots for native T1 and extracellular volume fraction within the interventricular septum. Bland-Altman plots for native T1 was shown in panel **a** and for extra cellular volume (ECV) in panel **b**. Open circles depict patients in whom MOLLI was acquired during breathholding. Coefficient of variation, COV; standard deviation, SD

Bland-Altman plots, along with mean bias, coefficient of variation, standard deviation and limits of agreement of interobserver variability for native T1 times and ECV in the IVS are shown in Fig. 7.

### Association between histological and CMR-derived fibrosis markers and clinical parameters

No correlation existed between CVF and heart age at the time of CMR or between either native T1 values or ECV and heart age. At the time of CMR, no patient had clinically significant rejection as per EmBx on the same day (ISHLT grade 0 R; *n* = 9; grade 1 R; *n* = 11). Seven patients (35%) had had at least one previous episode of acute cellular rejection (ACR) with an ISHLT grade ≥ 2 R. No differences in CVF, native T1 time, or ECV were identified between patients with and without previous 2 R ACR (*p* = 0.78). One patient had experienced an episode of antibody-mediated rejection 2.8 years after transplantation and 2.2 years before CMR, requiring extracorporeal membrane oxygenation and intensification of immunosuppression. This patient had the second highest CVF of all patients with 15.2% and highest native T1 time with 1070 ms, both in the IVS.

The authors’ center routinely performs ABO incompatible HTx in infants and young children. Five patients out of the eight patients who had undergone HTx before their second birthday had donor/recipient ABO incompatibility. No differences in CVF, native T1 time, or ECV were observed between patients with and without ABO miss-match.

No correlation existed between organ ischemia time at the time of HTx and CVF. Native T1 time, but not ECV, in the IVS correlated with ischemia time (*r* = 0.46, *p* < 0.05).

The six patients on antihypertensive medications did not have different CVF, native T1 or ECV in any of the investigated regions (IVS and entire LV) as compared to normotensive patients (*p* = 0.12 for CVF, *p* = 0.63 for native T1 in IVS, *p* = 0.51 for native T1 in entire LV, *p* = 0.98 for ECV in IVS, and *p* = 0.91 for ECV in entire LV respectively). No significant difference existed in LV mass between patients with and without antihypertensive medications (69 ± 18 g/m^2^ vs 63 ± 4 g/m^2^, *p* = 0.43).

Four patients underwent CMR within the first 90 days after HTx (median duration, 23 days; range, 9–51 days). The ‘early’ group did have higher ECV in the IVS and the entire LV, while CVF was not different in these 4 patients as compared to the other HTx patients who were investigated at least three months after HTx. (Table 3).

**Table 3:** Histological degree of fibrosis, native T1 time, and ECV in patients early and later after transplantation

		Early (<3 months) after HTx	Remaining	*p*
		*n* = 4	*n* = 16	
Collagen volume fraction, %		9.9 ± 1.7	10.0 ± 0.9	0.93
Native T1, msec	IVS	1022 ± 37	1005 ± 31	0.37
	Entire LV	1017 ± 29	985 ± 33	0.10
	Blood	1529 ± 74	1512 ± 81	0.67
ECV	IVS	0.33 ± 0.04	0.29 ± 0.03	<0.05
	Entire LV	0.32 ± 0.04	0.29 ± 0.03	0.05

Data are expressed as mean and standard deviation

*Abbreviations*: *ECV* extracellular volume fraction, *HTx* heart transplantation, *IVS* interventricular septum, *LV* left ventricle

No correlation was observed between N-terminal-pro-brain-natriuretic peptide and CVF, native T1 or ECV in the patient group.

Coronary angiography was not performed in close proximity to CMR and EmBx for ethical reasons. However, 15 patients underwent coronary angiography after CMR (median duration, 3.0 years; range 1.0– 4.6 years). One patient had ISHLT grade 1 CAV 1 year after CMR and deceased 1.5 years after coronary angiography. Another patient demonstrated ISHLT grade 2 CAV three years after CMR. The remaining 13 patients had normal coronaries at the time of coronary angiography.

### Relationship of CMR myocardial strain with histological and CMR-derived fibrosis markers

Global LV radial strain and strain rate were higher in HTx patients compared to controls (*p* < 0.0001 and *p* < 0.0005, respectively, Table 2). No differences in global LV longitudinal and circumferential strain and strain rate were identified between patients and controls. (Table 2) No association existed in the patients between any of the global strains or strain rates and CVF, native T1 or ECV measured within the entire LV and IVS.

### Relationship of ventricular size and function with histological and CMR-derived fibrosis markers

The results of ventricular size, mass, and ejection fraction analysis are summarized in Table 2. Patients after HTx had higher LV mass and LV mass-to-volume ratio as compared to controls. (Table 2) Thirteen patients demonstrated an LV ejection fraction below 55%, albeit under general anaesthesia. Eight patients had a right ventricular (RV) ejection fraction below 45%. No difference existed in CVF, native T1 times and ECV between patients with and without reduced CMR LV or RV ejection fraction. Left ventricular mass, as well as the ratio of LV mass to enddiastolic volume, showed no correlation with CVF, native T1 time or ECV. During a follow-up period of 7.8 ± 2.9 years, no patient underwent re-HTx and three patients died. One of them, who died from post-transplant lymphoproliferative disorder, was also the only one who had suffered from severe antibody-mediated rejection before CMR with the second highest CVF and highest septal native T1 time. The remaining two patients who’s CVF, native T1 time and ECV were below the cohort mean died from rejection or renal failure.

## Discussion

Despite a variety of candidate etiologies the morphological substrate for graft dysfunction following HTx is incompletely understood. Adverse fibrotic myocardial remodeling is a suspected longterm sequelae of HTx. However, until recently, it has not been feasible to quantify myocardial fibrosis non-invasively. The present study adds the following to our understanding of myocardial health in paediatric patients after HTx:

1. Native T1 time and ECV in the IVS correlate with histological degree of fibrosis in EmBx specimens.
2. Septal native T1 times are higher in patients with longer ischemia times at the time of transplantation.

Both ECV and native T1 times have been validated against the histological gold standard in adults with valvular heart disease, as well as ischemic and non-ischemic cardiomyopathy [16, 21, 23–25]. This is the first histological validation of markers of diffuse myocardial fibrosis by CMR relaxometry in children and the first study to validate ECV and T1 against CVF by RV septal EmBx in any age group. The correlations between histological CVF and native T1 times as well as ECV were modest. The level of agreement is in keeping with a study of postcontrast T1 vs. CVF in adult heart failure patients who underwent RV septal EmBx [22]. Studies in adults with myocardial biopsies from the LV, either by EmBx [26], intraoperative deep myocardial needle biopsy [21, 23, 25], surgical sampling [16] or analysis of the entire heart at the time of death or retransplantation [24], showed better agreement between CMR and histology than for the RV. The weaker agreement between RV tissue and LV T1 times and ECV is not surprising as there may be differences between RV and LV fibrosis and an RV septal sample may not be representative of LV myocardium [35]. In fact, one of the few studies which quantified ECV in congenital heart disease found higher RV than LV ECV in the same individual [36]. Some experts argue that findings on EmBx, right or left, may not be a representative of fibrotic changes found ‘deep’ within the allograft of end-stage post-transplant paediatric patients [6]. There are other potential explanations for the sub-optimal correlation between EmBx and CMR in our study and the one by Sibley and colleagues [22]: Although we strove to exclude all previous biopsy sites from analysis there remains the possibility that scarring from earlier procedures may have affected CVF quantification. Furthermore, the small size of the EmBx specimens may have served to reduce their representative value for the entire myocardium, despite our attempts to limit the effects of the small tissue size: CVF was assessed across each entire specimen, using automated quantification systems and uniform magnification, in contrast to earlier studies [16, 21, 23, 24]. It is interesting to note that native T1 times and ECV showed moderate correlation with CVF only in the IVS, but not for the entire LV. This may suggest that a) EmBx from the RV aspect of the interventricular septum are more reflective of the IVS than other parts of the LV, and that b) there is heterogeneity with regards to the degree of fibrotic remodeling throughout the LV. In fact, previous studies showed substantial segmental variation in T1 values and ECV [24, 37, 38]. It must be remembered that both T1 and ECV by CMR are only approximations of ‘fibrosis’ amounts and that both are affected by other myocardial processes, including edema.

In the present study as in other validation studies, ECV measured by CMR was higher than the histological CVF. Picrosirius red stains collagens which are the most abundant macromolecules in extracellular matrix. On the other hand, ECV depends on the uptake of gadolinium in the entire extracellular space. As such, it is a marker for the relative size of the extracellular matrix which contains other proteins like elastin, adhesive glycoproteins and proteoglycans in addition to collagen, as well as vascular volumes. As a result, although there is an association between the two ECV exceeds CVF. The average ECV in our paediatric controls was 0.22 ± 0.03, as compared to 0.25 ± 0.04 in adults [39]. These levels are in in keeping with in vivo studies using extracellular markers including ^14^C inulin or ^14^C mannitol in a rat model [40].

We demonstrated that ECV was significantly higher in patients early after HTx as compared to remaining patients. Exposure to cardiopulmonary bypass, reperfusion injury of the organ and other perioperative factors as well as possible episodes of early ACR are all factors which potentially promote myocardial edema as well as fibrotic remodeling [41].

Fibrosis within cardiac allografts is a well-documented phenomenon in adults [42, 43]. The results of increased septal native T1 time and ECV in children after HTx as compared to controls suggests that the same is true for paediatric patients. In a study evaluating serial EmBxs in 50 post-transplant adult patients, Armstrong et al. [43] showed that myocardial fibrosis develops as early as two months after transplant. Gramley et al. observed that fibrotic bundles form soon after HTx [44]. These two studies indicate that peri-transplant factors play a role in initiating a profibrotic cascade. Inflammation, recurrent episodes of rejection, metabolic disturbances and CAV [45] have all been proposed as promotors of adverse fibrotic remodeling. In the current study, septal T1 times were associated with longer graft ischemia times suggesting that minimizing them may lead to improved graft health [4, 46]. This association is in keeping with a correlation of cardiopulmonary bypass and aortic cross-clamp times with ECV in patients after Tetralogy of Fallot repair [47]. Larger prospective studies with a longitudinal component are necessary to answer the prognostic value of ECV and native T1.

We did not observe a relationship between LV or RV ejection fraction and histological or CMR markers of fibrosis, however, ejection fraction is a relatively insensitive marker of a decline in myocardial function [48]. Children and adolescents after HTx had reduced global radial strain and strain rate as compared to controls using CMR. The reduction in longitudinal strain in HTx patients was not significant. Of note, images for longitudinal strain analysis were available in only 9 controls, limiting the power of this comparison. This finding corroborates a report by Eleid et al. [49] who found reduced radial strain by speckle tracking in adult HTx patients irrespective of rejection. Similarly, Kailin and colleagues, also by speckle tracking [50], reported abnormal longitudinal, but not circumferential strain in paediatric HTx patients. Their study did not investigate radial strain. There was no association with CMR and histological myocardial fibrosis markers and strain or strain rate in the present study.

Bland-Altman analyses revealed only a small bias but relatively wide limits of agreements between the two observers. Breathholds were used for the MOLLI acquisition in 30% of patients, and a motion correction algorithm was not available at the time of data acquisition. In fact, Fig. 7 demonstrates that agreement was better for patients who were scanned during with breathholds, particularly for native T1.

### Limitations

First, the modest sample size of this pilot study may have obscured further differences between controls and patients as well as associations between fibrosis markers and other parameters. Second, T1 and ECV were measured on a single short axis slice and the assumption that these values are representative of the LV myocardium from base to apex may be erroneous. Third, it is possible that the difference in T1 and ECV between patients and controls was amplified by the lower hematocrit levels in HTx recipients, given the linear relationship between lower hematocrit and higher T1 and due to the fact that all myocardial regions of interest also sample small volumes of blood within the intramyocardial capillaries. However, if hematocrit, and not expansion of the extracellular space, was the primary determinant of T1, one would expect a correlation between hematocrit and native myocardial T1. No association between T1 and hematocrit was present, in the patient cohort. Furthermore, when comparing patients in the highest T1 quartile with those in the lowest T1 quartile we did not see a difference in hematocrit. Therefore, we do not believe that the differences in T1 and ECV are entirely or mainly due to variations in hematocrit. Finally, coronary angiography was not available in close proximity to CMR and EmBx for ethical reasons. Therefore, CAV, one of the candidate etiologies of fibrotic myocardial remodeling, could not be investigated.

## Conclusion

Native T1 time and ECV correlate with histologically determined collagen volume in paediatric patients after HTx. The graft organs in children after HTx undergo accelerated myocardial fibrotic remodeling, and higher native T1 times are associated with longer ischemia times.

## Additional file

Additional file 1: The average of collagen volume fraction in 100 biopsies. (DOCX 19 kb)

## Abbreviations

ACR: Acute cellular rejection
CAV: Coronary allograft vasculopathy
CMR: Cardiovascular magnetic resonance
CVF: Collagen volume fraction
ECV: Extracellular volume fraction
EmBx: Endomyocardial biopsy
HTx: Heart transplantation
ISHLT: International society for heart and lung transplantation
IVS: Intraventricular septum
LGE: Late gadolinium enhancement
LV: Left ventricle
MOLLI: Modified look-locker inversion recovery
RV: Right ventricle
SSFP: Steady-state free-procession sequence
