# Supplementary material for: Histological validation of cardiovascular magnetic resonance T1 mapping markers of myocardial fibrosis in paediatric heart transplant recipients
Source: J Cardiovasc Magn Reson. 2017 Feb 1;19:10. doi: 10.1186/s12968-017-0326-x (PMC5286863; doi:10.1186/s12968-017-0326-x)
Supplement: Additional file 1: — The average of collagen volume fraction in 100 biopsies. (DOCX 19 kb) [file 12968_2017_326_MOESM1_ESM.docx]

The average of collagen volume fraction in 100 biopsies

|  | Number of biopsies | Collagen volume fraction (%) | | | | | |
| --- | --- | --- | --- | --- | --- | --- | --- |
|  |  | 1 | 2 | 3 | 4 | 5 | 6 |
| Patient 1 | 6 | 10.8 | 10.4 | 12.8 | 10.7 | 12.7 | 11.4 |
| Patient 2 | 5 | 12.5 | 16.9 | 12.7 | 11.6 | 10.7 | n.a. |
| Patient 3 | 6 | 18.1 | 7.0 | 9.0 | 9.2 | 10.8 | 6.5 |
| Patient 4 | 4 | 11.4 | 12.8 | 12.1 | 13.3 | n.a. | n.a. |
| Patient 5 | 6 | 11.9 | 14.5 | 12.9 | 17.7 | 17.2 | 16.8 |
| Patient 6 | 6 | 7.6 | 11.4 | 15.2 | 16.2 | 17.3 | 14.6 |
| Patient 7 | 6 | 7.6 | 3.2 | 6.7 | 10.2 | 5.9 | 10.3 |
| Patient 8 | 5 | 3.5 | 3.1 | 4.0 | 5.4 | 4.5 | n.a. |
| Patient 9 | 3 | 16.0 | 14.4 | 18.9 | n.a. | n.a. | n.a. |
| Patient 10 | 6 | 9.3 | 6.3 | 7.2 | 7.8 | 8.7 | 9.3 |
| Patient 11 | 4 | 9.4 | 11.3 | 10.3 | 13.6 | n.a. | n.a. |
| Patient 12 | 5 | 8.5 | 9.9 | 6.9 | 10.0 | 7.6 | n.a. |
| Patient 13 | 3 | 10.4 | 8.1 | 6.3 | n.a. | n.a. | n.a. |
| Patient 14 | 5 | 8.8 | 6.6 | 6.2 | 8.0 | 6.0 | n.a. |
| Patient 15 | 5 | 8.7 | 8.0 | 8.4 | 8.9 | 7.3 | n.a. |
| Patient 16 | 5 | 11.2 | 11.2 | 11.3 | 10.1 | 9.2 | n.a. |
| Patient 17 | 5 | 9.4 | 10.5 | 11.1 | 12.3 | 11.0 | n.a. |
| Patient 18 | 4 | 8.9 | 4.5 | 5.9 | 5.0 | n.a. | n.a. |
| Patient 19 | 6 | 9.7 | 14.0 | 11.2 | 13.7 | 16.2 | 12.4 |
| Patient 20 | 5 | 4.2 | 5.1 | 4.4 | 4.1 | 6.6 | n.a. |

Three to six biopsied samples were taken in each individual. Collagen volume fraction was averaged among all 100 biopsy specimens from each individual.
